# The Birmingham Urban Climate Laboratory—A high density, urban meteorological dataset, from 2012–2014

**DOI:** 10.1038/sdata.2016.38

**Published:** 2016-06-07

**Authors:** Elliott L. Warren, Duick T. Young, Lee Chapman, Catherine Muller, C.S.B. Grimmond, Xiao-Ming Cai

**Affiliations:** 1 School of Geography, Earth and Environmental Sciences, University of Birmingham, Edgbaston B15 2TT, UK; 2 Department of Meteorology, University of Reading, Reading RG6 6BB, UK

**Keywords:** Atmospheric science, Climate-change impacts, Climate change

## Abstract

There is a paucity of urban meteorological observations worldwide, hindering progress in understanding and mitigating urban meteorological hazards and extremes. High quality urban datasets are required to monitor the impacts of climatological events, whilst providing data for evaluation of numerical models. The Birmingham Urban Climate Laboratory was established as an exemplar network to meet this demand for urban canopy layer observations. It comprises of an array of 84 wireless air temperature sensors nested within a coarser array of 24 automatic weather stations, with observations available between June 2012 and December 2014. data routinely underwent quality control, follows the ISO 8601 naming format and benefits from extensive site metadata. The data have been used to investigate the structure of the urban heat island in Birmingham and its associated societal and infrastructural impacts. The network is now being repurposed into a testbed for the assessment of crowd-sourced and satellite data, but the original dataset is now available for further analysis, and an open invitation is extended for its academic use.

## Background & Summary

In 2014, 54% of the world’s population reside within urban areas. As the world becomes increasingly urbanised, inhabitants have directly and indirectly modified their local environment, changing the dominant land-use, producing additional heat and reducing natural ventilation^1–3^. In doing so, they inadvertently change the local radiation budget, creating an environment that increasingly favours the development of Urban Heat Islands (UHIs) where temperature differences of up to 10 °C can be recorded between urban and rural areas^4,5^. The compound effect of this UHI effect, along with intense heat waves, and indeed a changing climate has significant impacts on the public health of urban populations both directly^6–8^, and indirectly via highly coupled infrastructure groups such as energy, transport, sanitation and communication^9,10^.

As a consequence, there is a demand for urban meteorological research to better understand the heterogeneity of UHIs to inform and improve adaptation and mitigation strategies, thereby reducing the vulnerability of society in the future^11–13^. Weather and climate models (at a range of scales), are increasing in sophistication to assist in this, and are leading to significant improvements in urban estimate accuracies, both spatially and temporally^4,14,15^. However, there have traditionally been insufficient in situ urban meteorological observations by which to evaluate models effectively^14,16^. Often data for cities actually comes from outside the urban area^17^ and there are difficulties in ascertaining the true representativeness of the few sites which exist within city boundaries. Fortunately, a growing trend of Urban Meteorological Networks (UMN) deployments are changing this (e.g., Oklahoma Mesonet^18^; Helsinki testbed^19^; Open Air Laboratories^20^). However, there is generally still an inadequate number of networks at an appropriately high spatial density to fully facilitate high resolution modelling. This is often due to a number of factors such as security, cost, and difficulty in finding appropriate siting^16,21^.

The Birmingham Urban Climate Laboratory (BUCL) is a high-density UMN situated in Birmingham, UK comprised of a dense array of 84 air temperature sensors nested within a course array of 24 weather stations. The network was initially established as a testbed for urban observations and as an exemplar for urban sensor deployment. The aim was to create a high quality dataset for use in the investigation of the spatio-temporal structure of Birmingham’s urban heat island, investigate the links between observations and the surrounding urban morphology, and as a dataset for the evaluation of numerical models^21^. Additionally, given the experience gained by the researchers in deploying the network, a further strand of work emerged, ultimately leading to a standardized UMN metadata protocol. It focussed on collating disparate guidelines for best practices in observing and documenting urban stations and existing meteorological networks, in order to improve data quality and applicability between urban meteorological networks^22^.

## Methods

### Automatic weather stations—coarse array

**Figure 1:**
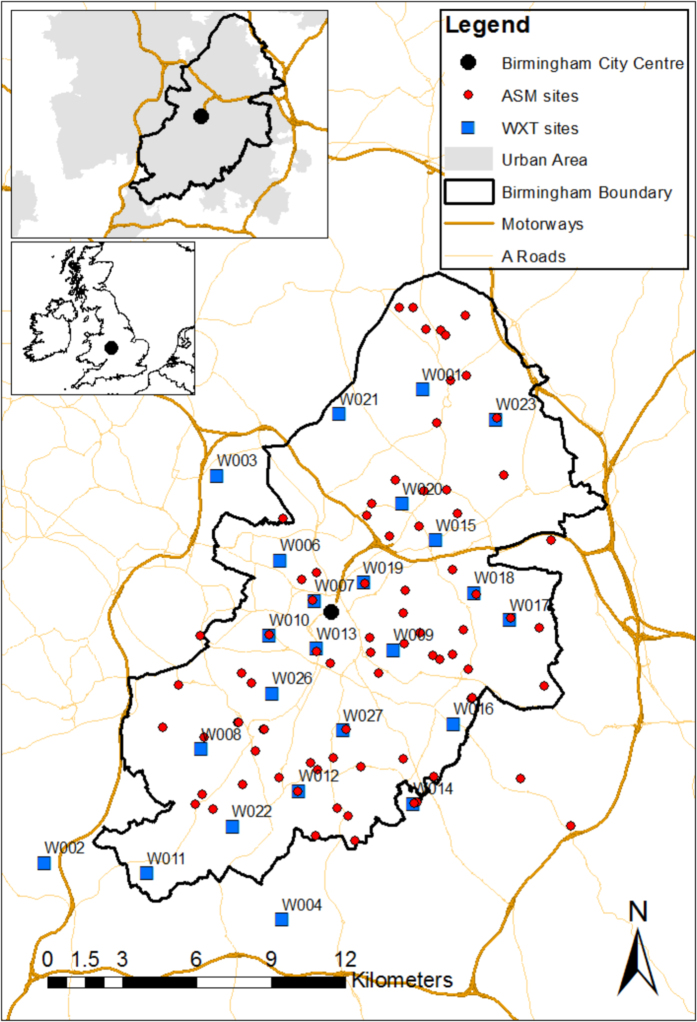
WXT and ASM deployment locations, between June 2012 and December 2014, within the Greater Birmingham area. Inset map of Birmingham with urban land use, and within the UK, in the top left of the map.

The coarse array consists of 24 Vaisala WXT520 (WXT) automatic weather stations, coupled with a Skye Instruments SKS1110 pyranometer, at minute resolution, with an average spacing of ~3 per km^2^ (Fig. 1). The full list of variables available for each station, and their sample types, is presented in Table 1. Each pair was equipped with a 12 V lead acid battery, trickle charged with a 10 W solar panel. Either a Campbell Scientific CR1000 with a Sierra Wireless Fastrack Xtend modem or a Campbell Scientific CR800 data logger with a COM110 modem was installed, facilitated by a SC105 data buffer which buffered data to the required bit rate. A GPRS antenna, and SIM card at a cost of £5 per month per site, was also required to enable communication. The central server used to store data from this network, was a Windows 7 personal computer, with a quad-core Intel i5 CPU, with 4096 MB RAM memory and an LSI PCI-SV92PP Soft Modem.

In the first 15 min of each hour, data collected by the WXTs was transmitted to a central server, using a Windows operating system, based at the University. This allowed the WXT to save power by reducing the time communication equipment was online. The specific time for each WXT to communicate was evenly distributed within the 15 min window, to reduce saturation of the server’s processor. A CRBasic program on the logger checks the remaining power and can limit communication attempts between hours, to further limit power use if necessary. Loggernet v4.3 software supplied by Campbell Scientific runs on the server, which archives raw data as Comma Separate Value (CSV) files for later processing. No further processing is done by Loggernet itself, but it does produce technical diagnostics, which inform the technician of each station’s current status and performance.

### Low-cost wireless temperature sensor—dense array

A high density array of 84 Aginova Sentinel Micro (ASM) air temperature sensors was deployed with an average spacing of ~1.5 km (Fig. 1). The sensor itself is a 10-kΩ negative temperature coefficient thermistor, supplied by a single AA 3.6 V 2.2 Ah Lithium-Thionyl chloride battery. All electronics were contained in a weather proof plastic enclosure. Each sensor was fitted with low-cost, bespoke, passively aspirated radiation shielding, which greatly reduced the impact of direct solar radiation^23^.

This array was innovative as it utilises existing Wi-Fi infrastructure via an Internet of Things (IoT) approach which allowed data to be collected from the decentralised network over the internet^24,25^. The sensors had an IEEE 802.11 b/g 2.4 GHz Wi-Fi communication card, allowing upload rates of up to 11 Mbps, an inbuilt flash memory capable of storing 1440 data points and two way sensor-server communication using User Datagram Protocol (UDP). As a result, the ASM was not dependent on an external logger or supporting communication equipment, such as with the WXTs. Thus, its overall size and cost were drastically reduced. A fuller description of the ASM is available in Young *et al.*^23^.

Wi-Fi compatibility allowed for free, near-real time data communication with the server through the schools network, for as long as a connection was maintained with a Wi-Fi access point. Upload frequency could also be flexibly altered to balance data upload and availability with battery power usage, which was based on individual sensor performance (set between 5 and 10 min in this network). Record lengths were mainly limited due to battery depletion, as a consequence of variable Wi-Fi stability and were visited when time allowed between other sensor deployments. Data, as documented in Table 2, were also transmitted to the Linux server at the University of Birmingham where it was transferred to a MySQL database by third-party software from Aginova, for later processing.

### Deployment

The majority of equipment is sited within the boundary of Birmingham (Fig. 1). In addition to the locations within the city, 3 AWS’s and 4 ASM’s were located at rural sites to provide background observations to aid the quantification of spatial variability of Birmingham’s UHI^21^. Efforts were made to ensure sensor locations were distributed to maximise the capture of the spatial variability of the UHI while accounting for the many different land use types within Birmingham.

Most of the equipment was located within schools, a deployment strategy chosen due to the large number of secure sites across the city and the availability of a Wi-Fi network to transmit data from the ASM. Climatologically, they have the added advantage of being surrounded by a variety of land use types/local climate zones ranging from dense urban, suburban and even light industrial^26^. Consequently, this helped maximise the representativeness of the observations taken, to the surrounding local-scale climate^16,27^. Transitional zones between land-use types, were avoided where possible.

In urban areas, a common compromise is often made in locating sensors on rooftops, which typically (unless located well above the roof surface) do not provide representative measurements at the local-scale due to the micro-scale processes^21,27^. In BUCL, sensors were installed at a height of 3±0.5 m, after consideration of sensor security and the need to place the sensor at a higher height due to the non-standard locations. It is accepted that the chosen sensor height is higher than the standard found in climatological sites (screen dry-bulb thermometer typically at 1.25 or 2 m). However urban sensor siting guidelines given by the World Meteorological Organisation (WMO) highlight, that previous studies have indicated, that there are negligible differences between observations made between 2 and 5 m in urban areas^17^. Diligence was observed to ensure that sensors were not sheltered by vegetation and a sufficient distance from buildings (at least 20 m to minimise anthropogenic heat and moisture impacting on the observations), in particular from the prevailing wind sectors. This ensured that winds observed were representative of the local area (sufficient fetch), that there was sufficient ventilation to the sensor within the radiation shield and to minimise the possible impact of micro-scale climates.

Detailed metadata were collected for all sites in accordance to the standardised UMN metadata protocol formulated by Muller *et al.*^22^. The availability of such metadata maximises the value of observations taken, with respect to understanding data representativeness and quality. This was recorded during sensor deployment and has been routinely updated during subsequent site visits, to maintain metadata relevance. Metadata includes, but is not limited to, location; elevation above sea level; micro and local scale sketch maps; cardinal direction photographs; panoramic photos at sensor height; surface and building types; and event horizons. For example, a compromise on optimal siting locations was necessary for some ASM locations, due to constraints pertaining to Wi-Fi signal strength and appropriate mounting locations. Where such compromises did take place, the metadata record provides the means to account for this during data analysis.

## Data Records

### Data format

**Figure 2:**
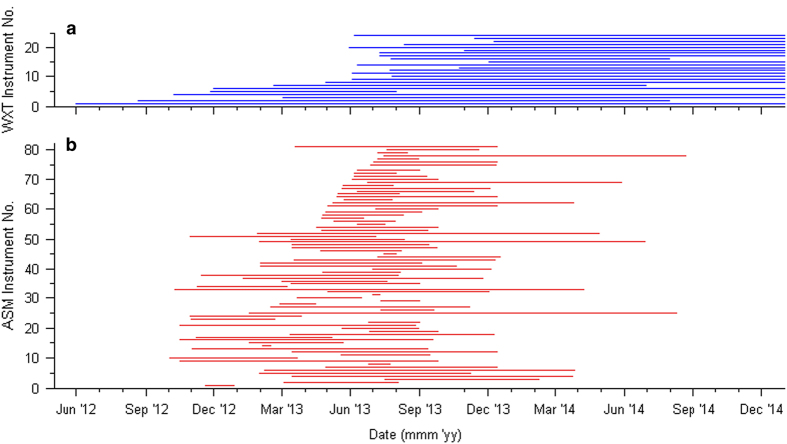
Data completeness over the BUCL UMN dataset period. (**a**) WXT records; (**b**) ASM records.

Data are available from the British Atmospheric Database Centre (BADC), and is divided into daily CSV files. WXT data is summarised monthly [Data Citation 1] where as ASM data are summarised daily [Data Citation 2]. File names follow the ISO8601 standard naming format: [*SID*_MinRes_*QAQC##*_Data_YYYY-MM-DD.csv], where *SID* is the name of the sensor as a four character string, with WXTs and ASMs beginning with W and S respectively, and *QAQC##* representing the level of quality control applied (See Technical Validation) e.g., [S004_MinRes_QAQC01_Data_2013-03-04.csv]. Figure 2 provides an illustration of data completeness within the dataset, to give an understanding of data richness throughout the full period.

Each file contains the header codes for all the variables observed, as presented in Tables 1 and 2. Importantly, the main header also contains the quality control stage each file was passed through, file date creation, WXT ID number and the district area the data were for. Quality Assurance/Quality Control is covered in detail in the Technical Validation section, but this is recorded in the data files in the final column for both WXT and ASM data. This takes the form of a summary composite flag, which is the highest flag issued for any measurement within the row. Each WXT data file has a corresponding data flag file within the *‘MinRes_QAQC02_NumericFlags’* directory, which contains the flags for each individual measurement. Data and flag files are constructed such that the location of data values and their corresponding flags, within the files, are the same.

### Metadata

Tabulated metadata for the WXTs and ASMs are provided on the standard template developed by Muller *et al.*^22^. It is available in the main directory entitled [WXT_Location_Elevation_Metadata.pdf] and [ASM_Location_Elevation.pdf]. Detailed metadata templates containing all the information collected, presented in Tables 1 and 2, is provided within the *‘WXT_Forms’* and *‘ASM_Forms’* directories. They all have standard filenames [UMN_Station_Metadata_*SID.*pdf] for easy identification. Metadata are ultimately more extensive for the WXTs due to the higher visitation frequency and therefore update opportunities.

## Technical Validation

### Equipment testing and routine visits

All WXTs passed testing at Vaisala in controlled conditions and were given independent test reports. Similarly, the coupled pyranometers were also initially tested in control conditions by Skye Instruments. After procurement, they were subject to an extended trial period on the Birmingham Urban Station Testbed (BUST) within the university weather station compound (Winterbourne No. 2 meteorological site). Up to four WXTs and pyranometers were deployed simultaneously on BUST which allowed for inter-comparison between the WXTs, and the UK Met Office/University of Birmingham meteorological equipment to determine the presence of any significant biases before deployment into the wider network array. BUST deployment periods varied between 2 and 10 weeks, depending on space availability on BUST and deployment opportunities in the wider network, as the time it took to organise siting with 3rd parties varied considerably in length.

The low-cost ASMs, were rigorously tested in environmental chambers at both the University of Birmingham and the UK Met Office. Mean errors of <±0.22 °C between −25 °C and 30 °C were detected in lab conditions against a platinum resistance thermometer (PRT). After calibration, field tests revealed mean errors <±0.24 °C and a RMSE of 0.13 °C, under all weather conditions, with the extra bespoke radiation shielding when compared to a PRT within a Stevenson screen. Full results of this testing are available in Young *et al.*^23^.

Routine maintenance visits continue to be made semi-annually to each WXT and a log is kept in the metadata of any changes to note. ASMs were visited less frequently, often for battery replacement, however this stopped as Wi-Fi problems ultimately prevented many sensors communicating and the remaining time and financial resources were focused on the WXTs. During each visit to either type of site, sensor shielding is cleaned, the site area generally tidied, the level of both the sensor and its fixings are checked, and metadata are updated. With the WXTs, silica bags are placed inside the enclosure to reduce moisture within it. These are also routinely replaced and checked for signs of moisture infiltrating the enclosure.

### Data quality control/ quality assurance

QA/QC is carried out daily through automated scripts using Mathworks MATLAB software. The full QA/QC tree is based on the approach used by the Oklahoma Mesonet, whereby erroneous data are not deleted but flagged and stored alongside it for future users to ultimately decide if it is suitable^28^. Table 3 shows the set of QA/QC filters used and Table 4 shows the flags that are assigned.

Failure of the relatively more objective tests, such as manufacture set instrumental limits, result in a severe flag being issued (2); other failures of relatively subjective tests results in a normal flag (1). Objective limit bounds for each variable were informed by equipment documentation and known atmospheric relations; with subjective limit bounds created from the technician’s experiences, informed by routine statistical analysis and experiences shared by other UMNs^28,29^. Missing data are found during the time check and is flagged (9), with place holders set to ‘−999’. After the flags have been issued, a summary flag is created to be included in the data file, which is the most severe flag assigned to any observation in the row.

After data were passed through the automated QA/QC scripts, stored data and flags have the identifier QAQC01 (signify automatic check) in the file name, setting it apart from the raw output. A final manual check is carried out on WXT data and their corresponding flags before their inclusion in the main BUCL UMN archive. This is to make any alterations to missed or wrongly applied flags, as well as identifying problems not picked up by the tests to limit the impact on future data quality. If multiple severe flags have been assigned, and an equipment failure has been identified, a failure flag (3) can be manually assigned. Good data found to be given a suspect (1) or warning (2) flag are reassigned with a ‘likely good’ (5) flag. This helps to separate it from automatically assigned good (0) data and could help signify issues with automatic tests. Once the manual check is completed, data are finally stored in the BUCL UMN archive with the identifier QAQC02 (automatic and manual check), ready for distribution.

It is important to note that the number of flags assigned for WXT data in winter is generally higher than other times of year. The latitude of Birmingham causes many of the WXTs to experience inadequate solar power to charge their battery and consequently make erroneous observations, often in the form of non-changing consecutive observation values. This is especially the case for wind, which has the largest power requirements (Table 1).

Data with warning (2) and failure (3) flags failed the more objective tests and should be omitted from use. Suspect data (1) are most likely erroneous but did not fail the stricter tests; consequently assigned the lesser flag. In order to minimise the inclusion of inaccurate data, the recommendation is to omit suspected data unless they are scrutinised further by end users and found to be valid. This was the case for rain and rainfall rate data which have been omitted for all WXTs except ‘W001’, and hail from all WXTs, due to problems with how the data were recorded, leading to an unreliable record.

## Usage Notes

Due to the compromise on optimal siting locations for some ASM locations, it is highly recommended to consult metadata, specifically sketch maps, photographs and event horizons, for each sensor to determine the potential impact it may have on its observational record.

## Additional Information

**How to cite this article:** Warren, E. L. *et al.* The Birmingham Urban Climate Laboratory—A high density, urban meteorological dataset, from 2012–2014. *Sci. Data* 3:160038 doi: 10.1038/sdata.2016.38 (2016).

## Supplementary Material



## Figures and Tables

**Table 1:** Headers used in the WXT files.

**Vaisala WXT520 (WXT)**				
**Column**	**Code**	**Variable**	**Units**	**Type**
1	YEAR	Year	—	TIME
2	MONX	Month	—	TIME
3	DAYX	Day	—	TIME
4	HOUR	Hour	—	TIME
5	MINX	Minute	—	TIME
6	TAIR	Air Temperature	degC	AVG
7	TDEW	Dew point Temperature	degC	CALC
8	RELH	Relative Humidity	%	AVG
9	PRES	Station Pressure	hPa	AVG
10	PSML	Mean Sea Level Pressure	hPa	CALC
11	SRAD	Solar Radiation	W m^−2^	AVG
12	RTOT	Rainfall Total	mm	TOT
13	RRAT	Rainfall Rate	kg m^−2^	CALC
14	WSPD	Wind Speed	m s^−1^	AVG
15	WDIR	Wind Direction	deg	AVG
16	WMAX	Maximum Wind Gust	m s^−1^	MAX
17	HAIL	Hail	Hits cm^2^	AVG
18	FLAG	Data Quality Control Flag	—	CALC
AVG -average; CALC—calculated; MAX—maximum; TOT—total. For averages, variables are sampled every 15 s and an average produced each minute; SMP—sample.				

**Table 2:** Headers used in the ASM files.

**Aginova Sentinel Micro (ASM)**				
**Column**	**Code**	**Variable**	**Units**	**Type**
1	YEAR	Year	—	TIME
2	MONX	Month	—	TIME
3	DAYX	Day	—	TIME
4	HOUR	Hour	—	TIME
5	MINX	Minute	—	TIME
6	TAIR	Air Temperature	degC	SMP
7	FLAG	Data Quality Control Flag	—	CALC
See Table 1 for further explanation of SMP, TIME or CALC.				

**Table 3:** List of all QA/QC tests currently carried out on the sensors (simplified version of Table 1 in Chapman *et al.*, 2014).

**QAQC Test**	**Sensors applied to**	**Flag if failed**	**Description**
Instrumental Range Test (i)	WXTs and ASMs	2	Test data against instrumental maximum and minimum tolerance bounds.
Range Test (Seasonal) (ii)	WXTs and ASMs	1	Range based on maximum and minimum plausible values, based on current season.
Paired Variable Check (i)	WXTs	2	Compare observations against each other based on established meteorological relationships, such as dry bulb temperature ≥wet bulb temperature ≥dew point temperature.
Time Check (i)	WXTs and ASMs	2 or 9	Identify duplicate (2) or missing (9) data and flag accordingly.
Spike and Dip (ii)	WXTs and ASMs	1	Maximum acceptable change and return to the original value within a defined time period.
Persistence Test (Runs) (i)	WXTs and ASMs	1	Maximum amount of time allowed for sensor observations not to change.
(i) Identifies tests always carried out; (ii) Extra tests that are carried out to explore potentially erroneous periods.			

**Table 4:** Flags used in the BUCL UMN QAQC process.

**Flag**	**Description**
0	Good
1	Suspect
2	Warning
3	Failure
5	Likely Good (BUCL Input)
9	Missing Data
